# The Back College for nurses – an evaluation of intermediate effects

**DOI:** 10.1186/s12995-019-0239-8

**Published:** 2019-06-20

**Authors:** Bianca Kusma, Aki Pietsch, Helge Riepenhof, Sören Haß, Daniel Kuhn, Klaus Fischer, Albert Nienhaus

**Affiliations:** 1 Institution for Statutory Accident Insurance in the Health and Welfare Services, Pappelallee 35/37, 22089 Hamburg, Germany; 2 BG Hospital Hamburg, Bergedorfer Straße 10, 21033 Hamburg, Germany; 3 BG Nordsee Reha-Klinik, Wohldweg 7, 25826 St. Peter-Ording, Germany; 4 BG Hospital Bergmannstrost Halle, Merseburger Straße 165, 06112 Halle (Saale), Germany; 5 Competence Centre for Epidemiology and Health Services Research for Healthcare Professionals (CVcare), University Medical Centre Hamburg-Eppendorf (UKE), Martinistrasse 52, 20246 Hamburg, Germany

**Keywords:** Nursing personnel, Low back pain, Occupational disease, Lumbar spine, Prevention

## Abstract

**Background:**

Nursing staff and care workers run an increased risk of work related musculoskeletal disorders such as low back pain. The Institution for Statutory Accident Insurance and Prevention in the Health and Welfare Services (BGW) offers its insured persons the opportunity to participate in a three-week Back College with the aim of preventing them having to abandon their profession due to back problems. The aim of the study was to record the effectiveness and sustainability of the Back College on an intermediate basis (6 months).

**Methods:**

As part of a single-group pre-post measurement on three survey dates – at the start (T0) and end (T1) of rehabilitation and 6 months later (T2) – in 2013 all participants in the Back College at three locations were surveyed using a standard questionnaire. Wilcoxon signed-rank tests were performed to evaluate statistically significant changes.

**Results:**

For measurement dates T0 to T2 we had 570 complete datasets (response rate 70.81%). There was a significant decrease in reported back pain and the general state of health and quality of life index improved. Participants’ emotional strain decreased and they showed an improved understanding of illness as well as of having acquired knowledge-based abilities and skills for dealing with the disease. After training, they recorded back-friendly behaviour in everyday life and opportunities to relieve strain on the spinal column were utilised at work more often. Participants’ subjective assessment of their ability to work (Work Ability Index) improved.

**Conclusion:**

The present study proved the intermediate effectiveness of the Back College curriculum. Whether these effects remain stable in the long term will be tested on the subsequent measurement date (T3, after 24 months).

## Introduction

The nursing profession must be viewed as a high-stress job due to numerous hazards, precarious working conditions, high workloads, long and irregular working hours, emotional pressures or understaffing for example [1]. Furthermore, the work of nurses is physically demanding. The job frequently involves heavy lifting, disadvantageous working postures, confined working environments, excessive manual forces, extended task duration, and high frequency/repetitions [2]. Their risk of experiencing back pain is increased [3–6] and the prevalence is higher than in other occupational groups [7]. The resulting medical impact is the cause of great discomfort and economic loss due to absence from work and lowered productivity [8, 9] as well as being a main reason for early retirement [10].

Considerable efforts have been made to prevent back problems, comprising low back pain education and awareness training [11, 12], education in patient handling with lifting techniques and back school [13, 14], ergonomic interventions and mechanical equipment [15–17], and individually designed physical training programs and stress management [18]. Reviews of occupational interventions have questioned the role of training in preventing job-related back pain [19, 20]. Due to the fact that back pain is complex and multifaceted, multidimensional interventions can be an appropriate approach. This assumption is consistent with previous findings [21, 22].

The Institution for Statutory Accident Insurance and Prevention in the Health and Welfare Services (BGW) is the provider of statutory accident insurance for non-state institutions in the health and welfare services in Germany. It is responsible for the prevention of workplace accidents and health hazards, rehabilitation, and workers’ compensation. In order to reduce the likelihood of nurses leaving their profession due to back problems, the BGW developed the Back College - an occupation-related secondary prevention measure.

### The Back college

This three-week inpatient training course was designed to reduce back pain and its risk factors in order to prevent OD 2108 (Occupational disease related to the vertebral discs of the lumbar spine). Based on a biopsychosocial approach, the Back College combines medical training therapy, physiotherapy and physical therapy with cognitive behavior modification elements and patient education provided by an interdisciplinary team.

As described earlier [23], the program is designed to increase physical fitness, including muscle strengthening exercises and cardiovascular conditioning, as well as to improve postural control. In addition, training is given occupation-specific practices. The objective is to develop and consolidate ergonomic principles in order to reduce lifting and carrying in patient-handling situations to a minimum. Participants are educated in proprioception, equilibrium and coordination. Practicing common, everyday movements is intended to consolidate new behavioral patterns. Psychological health training supports participants to cope with pain and stress. The course is rounded off with a lecture by a physician on anatomy and possible interference factors relating to the spine, nutritional advice, training in medical devices and aids, together with a lecture from a BGW representative on the legal prerequisites for OD 2108.

The BGW also offers follow-up support to its insured persons. Three months after taking part in the Back College or after their return to work, participants have the opportunity to receive workplace support by a therapist (e.g. sports medicine specialist or physiotherapist) over two days. This is intended to encourage implementation of the new techniques in everyday working routines. The therapist and employee work together to find solutions for performing different work routines economically in order to spare the back. Existing technical aids and transfer aids at the workplace are taken into account. The documentation of the workplace support forms the basis for the final discussions. Insured person, therapist as well as a BGW representative and the supervisor are taking part in order to ensure that necessary workplace changings are carried out.

Participants have also the possibility to attend a 5-day-refresher course. They can consolidate their acquired knowledge and solve questions and issues occurring during their daily working routine. This offer is free of charge and made between 12 and 18 month after attending the Back College.

The aim of the present study was to assess the effects of the Back College program on an intermediate basis (6 months).

## Methods

### Research design and participants

The present study was conducted in three hospitals of the Hospital Group of the Statutory Accident Insurance (BG Hospitals) offering the Back College program. As part of single group pre-post measurement at four times – at the beginning (T0) and end of rehabilitation (T1, 3 weeks after T0), and 6 months (T2) and 2 years later (T3) – all participants in 2013 were surveyed employing a standardized questionnaire.

The inclusion criteria were: insured person at the BGW, employed as a healthcare worker and exposure to heavy lifting at the workplace as well as work related low back pain or lumboischialgia. The exclusion criteria were: inadequate understanding of the German language, age below 18, severe visual or hearing impairment, and poor state of health.

Patients were recruited for the study in an introductory session on their first day at the Back College. They were informed both orally and in writing about the study purpose and procedure, and of their rights as participants.

Insured persons who met the inclusion criteria and gave informed consent to participate received the questionnaire and were asked to bring it back filled in the next day. Three weeks later they were issued with a second questionnaire, which was to be returned at the final discussion session. After 6 months, participants received the third questionnaire by post. Data collection took place from January 2013 to July 2014. In total, 570 of the 805 participants who received questionnaires returned them (response rate: 70.81%).

### Instrument

#### Socio-demographic data of respondents

Items on the questionnaire pertained to participants’ socio-demographic characteristics, such as gender, year of birth and family status. Details on professional background were also collected (i.e. educational specialism, years of experience).

#### Pain, drug use & sick leave due to lumbar spine

Respondents were asked to rate their current back pain on a numeric rating scale ranging from 0 (“no pain”) to 10 (“worst pain ever”). They were also asked to indicate how often they use drugs and were on sick leave due to lumbar spine issues.

#### Health-related quality of life

Health-related quality of life was assessed using the EuroQol [24] which consists of two parts, a descriptive system (EQ-5D-3 L) and a visual analogue scale (EQ-VAS). The EQ-5D-3 L covers the domains of mobility, self-care, usual activity, pain/discomfort and anxiety/depression. Patients were asked to rate their health on three levels of functioning (“no problems”, “some problems” or “extreme problems”) within each domain. The resulting five-digit number represents the respondent’s state of health (from “11111” meaning no problems at all to “33333” meaning extreme problems in all 5 domains). A total of 243 possible states of health are defined in which each state of health can be allocated to a certain value. Scores range from − 0.207 to 1, with negative values representing bad health states and 1 representing perfect health.

The visual analogue scale EQ VAS is used for assessment of participants’ health. It is thermometer-like and graded from 0 (representing “the worst health you can imagine”) to 100 (representing “the best health you can imagine”).

#### Physical activity

A modified version [25] of the Godin Leisure-Time Exercise Questionnaire [26, 27] was used to measure physical activity. This is a well-established reliable and valid measure for exercise behavior [28], that is often used in research of physical activity of patients with low back pain [29–32]. For these reasons the GLTEQ was applied in the present study.

Participants were asked to indicate the average number of sessions per week and average duration per week of strenuous (rapid heartbeats, sweating), moderate (not exhausting, light perspiration) and mild (minimal effort, no perspiration) physical activity in the past month. This applied only to activities outside of work duties (not business or at home). Responses (the product of frequency and duration) for each of these three activity categories were then computed.

#### Health education impact

The Health Education Impact questionnaire (heiQ™) [33, 34] was used to assess the impact of the Back College program on participants’ *emotional wellbeing* (α = 0.76), *self-monitoring and insight* (α = 0.65), and *skill and technique acquisition* (α = 0.72). This is a generic patient-reported measure of proximal outcomes of self-management programs. The questionnaire can be used across settings and disease groups. Answers are on a 4-point response scale (from “strongly disagree” to “strongly agree”). Scale values were calculated as the mean of values for the respective items, with higher scores implying better condition.

#### Program evaluation

The heiQ-programm™ (α = 0.65) was used to evaluate course quality in order to provide appropriate information about the quality of service delivery and satisfaction with the program. It consists of 9 items scored on a 6-point Likert scale (from “strongly disagree” to “strongly agree”). An average score between 1 and 6 can be calculated and a score of 5 or above indicates a clear positive result. A score of 4.5 or below can point to problems regarding course organisation or participants’ expectations.

#### Back postures in the daily routine

Using the modified version [35] of the motivation for spine-friendly behaviour questionnaire [36] (α = 0.92), insured persons were asked how often during the previous 2 weeks they had maintained an adequate body posture while performing activities in daily life such as sitting, standing, walking, lifting and carrying, with questions such as “sitting with a back-friendly posture”. Items were scored on a 5-point Likert scale (from “never” to “always”), with higher values indicating more back-friendly behaviour in everyday life.

#### Back postures in the working routine

In order to assess back postures adopted by participants during their daily working routine a questionnaire with 7 items was developed, with questions such as “creating space to work” or the “use of aids”. The reference period was 2 weeks. Answers were on 5-point scales (from “never” to “always”).

#### Work ability

The work ability of participants was assessed using the Work Ability Index (WAI). This questionnaire records the idea workers have of their own work ability. It comprises seven items including current work ability compared with lifetime best, work ability in relation to the demands of the job, the number of current diseases diagnosed by a physician, estimated work impairment due to diseases, sick leave during the past year (12 months), own prognosis of work ability 2 years from now, and mental resources [37]. The items were weighted and summed up to give a composite score of seven (poor work ability) to 49 (excellent work ability) [38]. The WAI score is classified in the following four categories: 7–27, poor; 28–36, moderate; 37–43, good; and 44–49, excellent.

#### Performance assessment and capacity testing

The participants’ perception of their capacity to perform activities in daily life was measured with the Performance Assessment and Capacity Testing Spinal Function Sort (PACT; [39, 40]. The PACT consists of 50 graphically depicted tasks with simple descriptions. Participants are asked to evaluate each task on a separate answer sheet on a 5-point scale from “able” to “restricted” to “unable”. The perceived functional ability scores range from 0 to 200. By categorising the scores according to work demands as defined by the Dictionary of Occupational Titles (DOT), it is possible to compare perceived functional ability [40]. Participants with a score <  100 were classified as having minimal working capacity (Table 1).

**Table 1:** Transformation of SFS scores to DOT categories [41, 42]

SFS score of perceived functional ability	Categories of work demands according to the DOT	T0	T1
< 100	Minimal work demands	117 (20.4%)	135 (23.6%)
100–124	Sedentary work (< 5 kg)	120 (20.9%)	81 (14.1%)
125–164	Light work (5–10 kg)	220 (38.4%)	195 (34%)
165–179	Medium work (10–25 kg)	49 (8.6%)	76 (13.3%)
180–194	Heavy work (25–45 kg)	33 (5.8%)	51 (8.9%)
> 195	Very heavy work (> 45 kg)	5 (0.9%)	9 (1.6%)
Missing		29 (5.1%)	26 (4.5%)

### Analysis

Frequency distributions were used to describe respondents’ demographic characteristics.

Non-responses to items were processed as missing data. The calculation of scale values was carried out as the mean of values. Due to the fact that the scores did not follow a normal distribution, Wilcoxon signed-rank tests were performed to evaluate statistically significant changes. Change scores in HeiQ constructs were evaluated as Cohen effect sizes (ES; change scores standardized using the pooled baseline SD) to measure the extent of changes in scores between the baseline and the two subsequent time points. ES = 0.1, ES = 0.3 and ES = 0.5 were considered to indicated small, medium and large changes, respectively.

All *p*-values given were two-tail. Statistical significance was set at *p* < 0.05. Values are given as mean and standard deviation (SD). Data was analysed using SPSS statistics version 22.

## Results

### Description of the cohort

The majority of participants were females (85.3%) with an average age of 48.84 years (SD = 8.03 years, range 20–63 years). With respect to qualifications, almost half of the participants were trained nurses (47.5%) followed by geriatric nurses (17.7%) and nursing assistants (11%). Most worked in inpatient facilities (78.1%). Half of the participants indicated that they work full time (54.4%). On average, participants had 24.7 years’ work experience (SD = 10.2 years). Table 2 summarizes the sociodemographic data.

**Table 2:** Description of the cohort

Variable	N (per cent)	M (SD)
Age		48.84 (8.03)
Gender (female)	486 (85.3%)	
Work experience (years)		24.74 (10.21)
Qualification		
Nursing	271 (47.5%)	
Geriatric nursing	101 (17.7%)	
Intensive care/OP/anaesthetics	55 (9.6%)	
Nursing assistant	63 (11%)	
Educator	27 (8.4%)	
Physiotherapist, occupational therapist	7 (1.2%)	
Other	43 (7.6%)	
Missing, non-response	3 (0.6%)	
Institution		
Inpatient facility	445 (78.1%)	
Semi-residential department	16 (2.8%)	
Outpatient facility	89 (15.6%)	
Surgery	3 (0.6%)	
Day care	10 (1.8%)	
Other	3 (0.6%)	
Missing, non-response	4 (0.7%)	
Hours worked per week		
Full time: 35 h and more	403 (54.4%)	
Part time: 15 to 34 h	324 (43.7%)	
Part time: under 15 h	12 (1.6%)	
Out of work	1 (0.1%)	
Missing, non-response	1 (0.1%)	

### Sick leave, drug use & back pain

The average number of 13.32 (SD = 32.63) days’ sick leave due to illness of the lumbar spine at T0 decreased to 5.49 (SD = 23.58) days at T2 (Z (*N* = 547) = − 7.43, *p* < .001). As depicted in Table 3 almost a quarter (21.8%) took medication once to twice a week and 16.8% on a daily basis at T0. Six months after the intervention (T2), the number of participants not taking medication increased from 25.5 to 41.9%. Sixteen percent took medication once to twice a week and 10% daily. The reported back pain decreased significantly between T0 and T2 (M_0_ = 3.86 vs M_2_ = 3.05; Z (*N* = 569) = − 9.275, *p* < .001).

**Table 3:** Drug use

Drug use	T0	T2
None	146 (25.5%)	240 (41.9%)
1 to 2 times in 6 months	54 (9.4%)	56 (9.8%)
1 to 2 times monthly	142 (24.8%)	119 (20.8%)
1 to 2 times a week	125 (21.8%)	95 (16.6%)
Daily	96 (16.8%)	60 (10.5%)
Missing, non-response	10 (1.7%)	3 (0.5%)

### Health-related quality of life

#### T0: health-related quality of life

Participants’ health-related quality of life as measured by the mean EQ-5D index value and EQ-VAS score was 0.82 ± 0.17 and 64.21 ± 17.48, respectively. A moderate positive correlation between the EQ-VAS and the EQ-5D index value (*r* = 0.37, *p* < .001) was found. The following distribution of ‘no problems’ across dimensions of QOL was reported: mobility 390 (68.1%), usual activities 320 (55.8%), self-care 561 (97.9%), pain/discomfort 51 (8.9%) and anxiety/depression 344 (60.0%). Participants reported 34 different states of health, in which 38 (6.6%) participants indicated no problems for any dimension, and two (0.4%), reported severe difficulty for all five dimensions.

#### T2: health-related quality of life

Six months after the Back College the mean EQ-5D index value and EQ-VAS score was 0.86 ± 0.15 and 70.71 ± 18.26, respectively. Both EQ-5D index value (Z (*N* = 561) = − 5.97. *p* < .001) and EQ-VAS (Z (*N* = 564) = − 8.718, *p* < .001) improved significantly from T0 to T2.

There was a moderate positive correlation between the EQ-VAS and the EQ-5D index value (*r* = 0.53, *p* < .001). Except for ‘self-care’ (*n* = 561; 97.9%), the number of participants who indicated ‘no problems’ across dimensions of QOL increased compared with T0: mobility 424 (74.0%), usual activities 383 (66.8%), pain/discomfort 129 (22.5%) and anxiety/depression 422 (73.6%). Thirty-one different states of health were reported, with 107 (18.7%) participants indicating no problems for any dimension, and one (0.2%) reporting severe difficulty for all five dimensions.

### Physical activity

At T0 the mean total physical activity was 4 h 22 min per week. Participants reported 1 h 7 min of strenuous, 1 h 51 min of moderate and 1 h 27 min of mild physical activity on average. Six months after Back College the reported total physical activity increased to 5 h 14 min per week. This difference is statistically significant (Z (*N* = 542) = − 5.26, *p* < .001). In detail, they indicated 1 h 23 min, 2 h 3 min and 1 h 48 min of strenuous, moderate and mild physical activity on average, respectively.

### Health education impact

#### T1: short-term changes in HeiQ constructs

At the end of rehabilitation and compared to baseline values a significant improvement and a large effect size was found in the *skill and technique acquisition* construct (ES = .62, *p* < .001). Small effect sizes were observed in *self-monitoring and insight* (ES = .19, *p* < .001) and *emotional wellbeing* (ES = .23, < .001).

#### T2: medium-term changes in HeiQ constructs

Six months after Back College and in comparison to baseline results, a large size effect was found in *skill and technique acquisition* (ES = .69, *p* < .001). Medium size effects were observed in *self-monitoring and insight* (ES = .39, *p* < .001) and *emotional wellbeing* (ES = .29, < .001). Both constructs showed medium improvements half a year later, although only small effect sizes were seen at T1.

### Work ability

At T0 the average WAI was 31.62 (SD = 6.48), ranging from 7 to 48. According to the WAI categorical classification, most of the participants had moderate (47.3%) or poor (25%) work ability. Twenty-three indicated good and only 1.7% excellent work ability.

Six months later 81 (14.1%), 211 (36.8%), 217 (37.9%), and 46 (8%) were in the poor, moderate, good, and excellent groups, respectively. The average WAI changed to 34.99 (SD = 6.90, range 7–49). This difference is statistically significant (Z (*N* = 554) = − 12.67, *p* < .001).

### Program evaluation

Participants’ perceptions of the Back College programme were largely positive (M = 5.57, SD = .43; Table 4). They intended to tell others that the “programme was worthwhile” and that “attending was worth their time and effort”. It is obvious that they felt supported by the programme leaders, who could handle difficult topics and were well organized. Participants also indicated that there was enough time to speak and that the group worked well together. On average, their responses approached “strongly agree” for most items.

**Table 4:** Mean scores on the heiQ™ programme evaluation questions

Questions about the Back College	Mean (SD)
I intend to tell other people that the programme is very worthwhile	5.83 (.43)
The programme has helped me to set goals that are reasonable and within reach	5.51 (.67)
I trust the information and advice I was given in the programme	5.59 (.58)
Course leaders were very well organized	5.28 (.84)
I feel it was worth my time and effort to take part in the programme	5.74 (.53)
Difficult topics and discussions were handled well by my programme leaders	5.47 (.71)
I thought the programme content was very relevant to my situation	5.30 (.74)
I feel that everyone in the programme had the chance to speak if they wanted	5.71 (.54)
The people in the group worked very well together	5.71 (.56)

Items were scored on a 6-point Likert scale from 1 = strongly disagree to 6 = strongly agree

### Back posture habits in working and daily routine

Half a year after attending Back College, participants recorded more back-friendly behaviour in everyday life (M_0_ = 3.02 vs M_2_ = 3.77; Z (*N* = 561) = −18.184, *p* < .001).

**Fig. 1:**
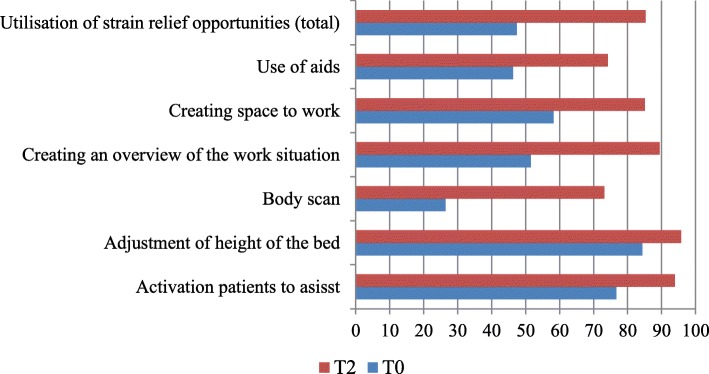
Utilisation of strain relief opportunities during everyday care (percentage of respondents who stated “frequently/always”)

Furthermore, they indicated that opportunities to relieve strain on the spinal column were more often utilised at work (Fig. 1).

### Performance assessment and capacity testing

At the beginning of the Back College the mean PACT score was low, at 129. According to the DOT categories of work demands (Table 1), most of the participants had the ability to perform light (38.4%), sedentary (20.9%) work or meet minimal work demands (20.4%). Nine percent reported a capability to perform medium work. And only 5.8 and 0.9% indicated an ability to do heavy or very heavy work respectively.

Three weeks later 135 (23.6%), 81 (14.1%), 195 (34.0%), 76 (13.3%), 51 (8.9%) and 9 (1.6%) were in the minimal, sedentary, light, medium, heavy, and very heavy work groups, respectively. The self-perception of capacity increased marginally, to 134.46 points on average. This difference is statistically significant (Z (*N* = 533) = − 3.69, *p* < .001).

## Discussion

The overall performance of participants was improved by training them to employ spine-friendly techniques at work, adequate movement behaviour in their daily routine and intensive muscle strengthening exercises. Half a year after their participation in the Back College programme, attendees reported significantly less pain. This result is consistent with earlier evaluation studies of the Back College. An improvement and reduction in pain intensity during the survey period was observed in studies by Kromark, Rojahn & Nienhaus [43] as well as Koch et al. [23]. A possible explanation for this result could be the individual increase in the level of activity as well as the appropriate application of knowledge acquired during the Back College about physical activity, effects and background information.

Specifically, knowledge about the different causes of back pain and its common and often harmless occurrence – especially in combination with the skills taught at the Back College, such as how to be able to manage one’s own pain and to actively address it – could have resulted in a changed perception of back pain.

In accordance with the literature, the programme, rather than approaching back pain as a serious disease, reinterprets it as simple episodes that can be managed independently by the patients themselves [44–47].

The programme’s success is further suggested by a reduction in the number of sick leave days and the use of medication due to back pain during the study period. This decrease in sick leave days could not be proven by Kromark et al. [43]. A possible explanation for this is the fact that in that study data was collected on average 26 months later and the participants may not have been able to accurately remember the length of the sick leave and the exact cause for it. In the current study, in contrast, the data collection period was only 6 months. The participants reported a high satisfaction with the Back College. This was also demonstrated in an earlier study [23].

Within the assessment period, perceived general health as well as quality of life improved. Consequently, by participating in the Back College training, the participants’ subjective perception and capacity to act in terms of physical, psychological and social aspects – those aspects that determine the health-related quality of life – could be influenced positively [48]. It can be assumed that the Back College training content, especially the teaching of knowledge and skills, has strengthened health resources and thus improved the perception of wellbeing too. The medium to high effect sizes of the heiQ scales after 6 months also confirm this. Back College participants acquire knowledge-based skills and strategies to better manage their back issues; they learn to monitor their health and set responsible goals and boundaries. Thus, the negative health-related aspect can be reduced as well. The improvement in perceived overall health in particular may be traced back to the reduction of back pain, the increase in physical activity and the increase in back-related knowledge. The experience that participants themselves can influence their own back health and can acquire competence to actively influence it at the Back College may have had an impact on the participants’ health-related satisfaction and quality of life.

Research has shown close relations between the perceived overall state of one’s health and satisfaction with life as well as locus of control [49]. Therefore, perceived health does not only reflect the physical health that can be objectively determined, but also overall life satisfaction and the belief that one’s health situation can be influenced through one’s own behavior.

In 3 weeks at the Back College, the PACT values of participants improved. Perceived self-efficacy is a important factor contributing to the outcome in patients with chronic back pain [50]. According to Bandura [51], perceived self-efficacy has an impact on how people behave in difficult situations, and people who doubt their capabilities shy away from tasks which they regard as personal threats. The back pain experienced in patient-handling situations may have affected participants’ perceived functional ability. Due to the intensive training at the Back College, participants’ perception of their individual physical performance may have changed as well. While at the beginning some restrictions were emphasized, over time participants developed better self-assessment of their effective capabilities.

Another goal of the Back College is to increase participants’ physical activity and muscle development. The training is intended to improve overall performance. A measurable increase in the physical activity of participants would suggest performance of physical activity in the individual’s daily routine. The aim of improving physical fitness is not only to reduce back pain but also to enable participants to adopt spine-friendly ergonomic movement habits in their occupational as well as their daily routines. The application of this was shown by earlier studies [23, 43] and the current study also demonstrates a high application rate. However, changes in muscle strength were not assessed in our study.

Research has shown that manual handling of patients is associated with a high lumbar load for nurses [52]. The risk of developing an intervertebral disc-related disease is considerably high in the profession. A review from Burdorf et al. [53] indicated that good implementation of lifting devices is required to noticeably reduce LBP and injury claims. Jaromi et al. [54] demonstrated that the combination of manual handling training and Back School achieved a significant reduction in pain intensity and improvement in body posture. Jäger et al. were able to show that by implementing appropriate working techniques a significant reduction in lumbar spinal loads can be achieved. If smaller aids such as a slide mat or a sliding board are used, the load on the employee’s back can be further reduced [52].

This could also have led to perceived improvements in the participants’ ability participants to work. In a study by Michaelis and Herman [55], significant positive effects on the perceptions of spinal loads, back issues and the overall ability to work were demonstrated. A prerequisite for this is the correct and regular application of spine-friendly working techniques, which can be embedded in participants’ occupational routines. Furthermore, the utilization of aids can reduce the risk of the occurrence of (lumbar) sciatica by half [55] as well as reducing physical loads [56].

In contrast, a recent review by Van Hoof et al. [57] found no strong evidence for any intervention for the prevention and treatment of low back pain in nurses. Isolated manual handling and stress management were not effective in nurses with and without low back pain. An additional stretching exercise programme yielded a better result than performing usual activities; the combination of manual handling training and back school was better than passive physiotherapy and a multidimensional intervention achieved no better results than a general exercise intervention in reducing low back pain in nurses. The authors conclude that multidimensional interventions may be more effective for nurses who already experience low back pain and not for primary prevention [57]. Moreover, interventions which are more specifically tailored to the needs of patients can enhance effectiveness [58]. The multimodal concept of the Back College is designed to meet the special needs of healthcare workers.

### Limitations

As with all empirical research, some limitations of the current study need to be mentioned. The study is questionnaire based. Therefore no functional tests were performed. This limitation should be considered when interpreting the results of the study. As the data is based on self-reports, participants may have felt obliged to answer in a socially acceptable manner. It is not known whether new techniques and a changed working routine are used in daily patient-handling situations. To fully examine this, an objective measurement would be useful. Direct [59] or indirect [60] field observations would give more accurate information about implementation and the use of transfer techniques.

Another limitation is the lack of a control group. Given the single group pre-post design, the changes found cannot be causally attributed to the effects of the Back College. Although a non-treatment group would be desirable, it is difficult to realize due to legal restrictions. According to the German Social Code the BGW has to make every effort to reduce the risk that its insured persons suffer the occurrence, recurrence or deterioration of an occupational disease.

## Conclusion

Despite the limitations of our study, our data support the assumption that the Back Colleges is effective in improving the back health of nurses. A long-term evaluation is warranted.

## Data Availability

The dataset supporting the conclusions of this article is included within the article.

## References

[1] Hammig O (2018). Explaining burnout and the intention to leave the profession among health professionals - a cross-sectional study in a hospital setting in Switzerland. BMC Health Serv Res.

[2] Choi SD, Brings K (2015). Work-related musculoskeletal risks associated with nurses and nursing assistants handling overweight and obese patients: a literature review. Work.

[3] Abedini R, Choobineh AR, Hasanzadeh J (2015). Patient manual handling risk assessment among hospital nurses. Work.

[4] Samaei SE, Mostafaee M, Jafarpoor H, Hosseinabadi MB (2017). Effects of patient-handling and individual factors on the prevalence of low back pain among nursing personnel. Work.

[5] Shieh SH, Sung FC, Su CH, Tsai Y, Hsieh VC (2016). Increased low back pain risk in nurses with high workload for patient care: a questionnaire survey. Taiwan J Obstet Gynecol.

[6] Genc A, Kahraman T, Goz E (2016). The prevalence differences of musculoskeletal problems and related physical workload among hospital staff. J Back Musculoskelet Rehabil.

[7] Harcombe H, Herbison GP, McBride D, Derrett S (2014). Musculoskeletal disorders among nurses compared with two other occupational groups. Occup Med (Lond).

[8] Olafsson G, Jonsson E, Fritzell P, Hagg O, Borgstrom F (2018). Cost of low back pain: results from a national register study in Sweden. Eur Spine J.

[9] Andersen LL, Clausen T, Burr H, Holtermann A (2012). Threshold of musculoskeletal pain intensity for increased risk of long-term sickness absence among female healthcare workers in eldercare. PLoS One.

[10] Jensen LD, Ryom PK, Christensen MV, Andersen JH. Differences in risk factors for voluntary early retirement and disability pension: a 15-year follow-up in a cohort of nurses’ aides. BMJ Open. 2012;2:e000991. 10.1136/bmjopen-2012000991. 10.1136/bmjopen-2012-000991 PMC3533112 23148337

[11] Karahan A, Bayraktar N (2013). Effectiveness of an education program to prevent nurses’ low back pain: an interventional study in Turkey. Workplace Health Saf.

[12] Kozak A, Freitag S, Nienhaus A (2017). Evaluation of a training program to reduce stressful trunk postures in the nursing professions: a pilot study. Ann Work Expo Health.

[13] Carta A, Parmigiani F, Roversi A, Rossato R, Milini C, Parrinello G, Apostoli P, Alessio L, Porru S (2010). Training in safer and healthier patient handling techniques. Br J Nurs.

[14] Jaromi M, Kukla A, Szilágyi B, Simon-Ugron A, Bobály VK, Makai A, Linek P, Ács P, Leidecker E (2018). Back school programme for nurses has reduced low back pain levels: a randomised controlled trial. J Clin Nurs.

[15] Hegewald Janice, Berge Wera, Heinrich Philipp, Staudte Ronny, Freiberg Alice, Scharfe Julia, Girbig Maria, Nienhaus Albert, Seidler Andreas (2018). Do Technical Aids for Patient Handling Prevent Musculoskeletal Complaints in Health Care Workers?—A Systematic Review of Intervention Studies. International Journal of Environmental Research and Public Health.

[16] Freiberg A, Euler U, Girbig M, Nienhaus A, Freitag S, Seidler A (2016). Does the use of small aids during patient handling activities lead to a decreased occurrence of musculoskeletal complaints and diseases? A systematic review. Int Arch Occup Environ Health.

[17] Dennerlein JT, O'Day ET, Mulloy DF, Somerville J, Stoddard AM, Kenwood C, Teeple E, Boden LI, Sorensen G, Hashimoto D (2017). Lifting and exertion injuries decrease after implementation of an integrated hospital-wide safe patient handling and mobilisation programme. Occup Environ Med.

[18] Jensen LD, Gonge H, Jors E, Ryom P, Foldspang A, Christensen M, Vesterdorf A, Bonde JP (2006). Prevention of low back pain in female eldercare workers: randomized controlled work site trial. Spine (Phila Pa 1976).

[19] Bos EH, Krol B, Van Der Star A, Groothoff JW (2006). The effects of occupational interventions on reduction of musculoskeletal symptoms in the nursing profession. Ergonomics.

[20] Martimo KP, Verbeek J, Karppinen J, Furlan AD, Takala EP, Kuijer PP, Jauhiainen M, Viikari-Juntura E (2008). Effect of training and lifting equipment for preventing back pain in lifting and handling: systematic review. BMJ.

[21] Rasmussen CD, Holtermann A, Bay H, Sogaard K, Birk Jorgensen M (2015). A multifaceted workplace intervention for low back pain in nurses’ aides: a pragmatic stepped wedge cluster randomised controlled trial. Pain.

[22] Shojaei S, Tavafian SS, Jamshidi AR, Wagner J (2017). A multidisciplinary workplace intervention for chronic low Back pain among nursing assistants in Iran. Asian Spine J.

[23] Koch P, Pietsch A, Harling M, Behl-Schon S, Nienhaus A (2014). Evaluation of the Back college for nursing staff. J Occup Med Toxicol.

[24] The EuroQol Group (1990). EuroQol-a new facility for the measurement of health-related quality of life. Health Policy.

[25] Lippke S, Ziegelmann JP, Schwarzer R, Velicer WF (2009). Validity of stage assessment in the adoption and maintenance of physical activity and fruit and vegetable consumption. Health Psychol.

[26] Godin G, Shephard RJ (1985). A simple method to assess exercise behavior in the community. Can J Appl Sport Sci.

[27] Plotnikoff RC, Lipke S, Reinbold-Matthews M, Courneya KS, Karunamuni N, Sigal RJ, Birkett N (2007). Assessing the validity of a stage measure on physical activity in a population-based sample of individuals with type 1 or type 2 diabetes. Meas Phys Educ Exerc Sci.

[28] Garcia D, Daniele T, Archer T (2017). A brief measure to predict exercise behavior: the Archer-Garcia ratio. Heliyon.

[29] Burns SA, Cleland JA, Rivett DA, Snodgrass SJ (2018). Effectiveness of physical therapy interventions for low back pain targeting the low back only or low back plus hips: a randomized controlled trial protocol. Braz J Phys Ther.

[30] Shebib R, Bailey JF, Smittenaar P, Perez DA, Mecklenburg G, Hunter S (2019). Randomized controlled trial of a 12-week digital care program in improving low back pain. npj Digital Medicine.

[31] Welch N, Moran K, Antony J, Richter C, Marshall B, Coyle J, Falvey E, Franklyn-Miller A (2015). The effects of a free-weight-based resistance training intervention on pain, squat biomechanics and MRI-defined lumbar fat infiltration and functional cross-sectional area in those with chronic low back. BMJ Open Sport Exerc Med.

[32] Sutherlin MA, Gage M, Mangum LC, Hertel J, Russell S, Saliba SA, Hart JM (2018). Changes in muscle thickness across positions on ultrasound imaging in participants with or without a history of low Back pain. J Athl Train.

[33] Osborne RH, Elsworth GR, Whitfield K (2007). The health education impact questionnaire (heiQ): an outcomes and evaluation measure for patient education and self-management interventions for people with chronic conditions. Patient Educ Couns.

[34] Schuler M, Musekamp G, Faller H, Ehlebracht-Konig I, Gutenbrunner C, Kirchhof R, Bengel J, Nolte S, Osborne RH, Schwarze M (2013). Assessment of proximal outcomes of self-management programs: translation and psychometric evaluation of a German version of the health education impact questionnaire (heiQ). Qual Life Res.

[35] Meng K, Seekatz B, Roband H, Worringen U, Vogel H, Faller H (2011). Intermediate and long-term effects of a standardized back school for inpatient orthopedic rehabilitation on illness knowledge and self-management behaviors: a randomized controlled trial. Clin J Pain.

[36] Herda C, Keller S, Ridder K, Basler H-D. Motivation for spine-friendly behavior. [Original title: Motivation zu rückenfreundlichem Verhalten.]. In Motivation to behavior change [Original title: Motivation zur Verhaltensänderung]. Edited by Keller S. Freiburg i. B. Lambertus; 1999.

[37] Ilmarinen J (2007). The work ability index (WAI). Occup Med (Lond).

[38] Roelen CA, Heymans MW, Twisk JW, van der Klink JJ, Groothoff JW, van Rhenen W (2014). Work ability index as tool to identify workers at risk of premature work exit. J Occup Rehabil.

[39] Matheson LN, Matheson ML (1989). Spinal function Sort: rating of perceived capacity. Text booklet and Examiner's manual. Trabuco canyon: performance assessment and capacity testing.

[40] Matheson LN, Matheson ML, Grant J (1993). Development of a measure of perceived functional ability. J Occup Rehabil.

[41] Oesch PR, Hilfiker R, Kool JP, Bachmann S, Hagen KB (2010). Perceived functional ability assessed with the spinal function sort: is it valid for European rehabilitation settings in patients with non-specific non-acute low back pain?. Eur Spine J.

[42] Trippolini MA, Dijkstra PU, Geertzen JH, Reneman MF (2015). Measurement properties of the spinal function Sort in patients with sub-acute whiplash-associated disorders. J Occup Rehabil.

[43] Kromark K, Rojahn K, Nienhaus A (2005). Disc-induced pathologies of the lumbar spine in nurses. Trauma Berufskrankh.

[44] Darlow B, Dowell A, Baxter GD, Mathieson F, Perry M, Dean S (2013). The enduring impact of what clinicians say to people with low back pain. Ann Fam Med.

[45] Darlow B, Perry M, Stanley J, Mathieson F, Melloh M, Baxter GD, Dowell A (2014). Cross-sectional survey of attitudes and beliefs about back pain in New Zealand. BMJ Open.

[46] Wertli MM, Rasmussen-Barr E, Held U, Weiser S, Bachmann LM, Brunner F (2014). Fear-avoidance beliefs-a moderator of treatment efficacy in patients with low back pain: a systematic review. Spine J.

[47] Glattacker M, Heyduck K, Meffert C (2013). Illness beliefs and treatment beliefs as predictors of short-term and medium-term outcome in chronic back pain. J Rehabil Med.

[48] Koller M, Neugebauer EA, Augustin M, Bussing A, Farin E, Klinkhammer-Schalke M, Lorenz W, Munch K, Petersen-Ewert C, Steinbuchel N, Wieseler B (2009). Assessment of quality of life in health services research - conceptual, methodological and structural prerequisites. Gesundheitswesen.

[49] Infurna FJ, Gerstorf D, Ram N, Schupp J, Wagner GG (2011). Long-term antecedents and outcomes of perceived control. Psychol Aging.

[50] Keedy NH, Keffala VJ, Altmaier EM, Chen JJ (2014). Health locus of control and self-efficacy predict back pain rehabilitation outcomes. Iowa Orthop J.

[51] Bandura A, Ramachaudran V (1994). Self-efficacy. In encyclopedia of human behavior.

[52] Jager M, Jordan C, Theilmeier A, Wortmann N, Kuhn S, Nienhaus A, Luttmann A (2013). Lumbar-load analysis of manual patient-handling activities for biomechanical overload prevention among healthcare workers. Ann Occup Hyg.

[53] Burdorf A, Koppelaar E, Evanoff B (2013). Assessment of the impact of lifting device use on low back pain and musculoskeletal injury claims among nurses. Occup Environ Med.

[54] Jaromi M, Nemeth A, Kranicz J, Laczko T, Betlehem J (2012). Treatment and ergonomics training of work-related lower back pain and body posture problems for nurses. J Clin Nurs.

[55] Michaelis M, Hermann S (2010). Evaluation of the nursing concept “Back protective patient transfer”.

[56] European Commission (2011). Occupational health and safety risks in the healthcare sector. Guide to prevention and good practice.

[57] Van Hoof W, O'Sullivan K, O'Keeffe M, Verschueren S, O'Sullivan P, Dankaerts W (2018). The efficacy of interventions for low back pain in nurses: a systematic review. Int J Nurs Stud.

[58] Kamper SJ, Apeldoorn AT, Chiarotto A, Smeets RJ, Ostelo RW, Guzman J, van Tulder MW (2015). Multidisciplinary biopsychosocial rehabilitation for chronic low back pain: Cochrane systematic review and meta-analysis. BMJ.

[59] Karstad K, Rugulies R, Skotte J, Munch PK, Greiner BA, Burdorf A, Sogaard K, Holtermann A (2018). Inter-rater reliability of direct observations of the physical and psychosocial working conditions in eldercare: an evaluation in the DOSES project. Appl Ergon.

[60] Warming S, Juul-Kristensen B, Ebbehoj NE, Schibye B (2004). An observation instrument for the description and evaluation of patient transfer technique. Appl Ergon.

